# Membrane estrogen receptor α signaling modulates the sensitivity to estradiol treatment in a dose- and tissue- dependent manner

**DOI:** 10.1038/s41598-023-36146-9

**Published:** 2023-06-03

**Authors:** Yiwen Jiang, Karin Horkeby, Petra Henning, Jianyao Wu, Lina Lawenius, Cecilia Engdahl, Priti Gupta, Sofia Movérare-Skrtic, Karin H. Nilsson, Ellis Levin, Claes Ohlsson, Marie K. Lagerquist

**Affiliations:** 1 Sahlgrenska Osteoporosis Centre, Centre for Bone and Arthritis Research at Institute of Medicine, Sahlgrenska Academy at University of Gothenburg, Vita Stråket 11, S-413 45 Göteborg, Sweden; 2 Department of Drug Treatment, Sahlgrenska University Hospital, Region Västra Götaland, Gothenburg, Sweden; 3 Department of Rheumatology and Inflammation Research, Institute of Medicine, Sahlgrenska Academy at University of Gothenburg, Gothenburg, Sweden; 4 Division of Endocrinology, Department of Medicine, University of California, Irvine, Irvine, CA 92697 USA; 5 Department of Veterans Affairs Medical Center, Long Beach, Long Beach, CA 90822 USA

**Keywords:** Hormone receptors, Physiology

## Abstract

Estradiol (E2) affects both reproductive and non-reproductive tissues, and the sensitivity to different doses of E2 varies between tissues. Membrane estrogen receptor α (mERα)-initiated signaling plays a tissue-specific role in mediating E2 effects, however, it is unclear if mERα signaling modulates E2 sensitivity. To determine this, we treated ovariectomized C451A females, lacking mERα signaling, and wildtype (WT) littermates with physiological (0.05 μg/mouse/day (low); 0.6 μg/mouse/day (medium)) or supraphysiological (6 μg/mouse/day (high)) doses of E2 (17β-estradiol-3-benzoate) for three weeks. Low-dose treatment increased uterus weight in WT, but not C451A mice, while non-reproductive tissues (gonadal fat, thymus, trabecular and cortical bone) were unaffected in both genotypes. Medium-dose treatment increased uterus weight and bone mass and decreased thymus and gonadal fat weights in WT mice. Uterus weight was also increased in C451A mice, but the response was significantly attenuated (− 85%) compared to WT mice, and no effects were triggered in non-reproductive tissues. High-dose treatment effects in thymus and trabecular bone were significantly blunted (− 34% and − 64%, respectively) in C451A compared to WT mice, and responses in cortical bone and gonadal fat were similar between genotypes. Interestingly, the high dose effect in uterus was enhanced (+ 26%) in C451A compared to WT mice. In conclusion, loss of mERα signaling reduces the sensitivity to physiological E2 treatment in both non-reproductive tissues and uterus. Furthermore, the E2 effect after high-dose treatment in uterus is enhanced in the absence of mERα, suggesting a protective effect of mERα signaling in this tissue against supraphysiological E2 levels.

## Introduction

Estradiol (E2) is a steroid sex hormone with crucial pleiotropic effects in multiple organs in both females and males. Via estrogen receptor signaling, E2 regulates reproductive activities, cardiovascular physiology, bone homeostasis, lipid metabolism, etc.^1–4^. The concentration of E2 decreases with age, especially in women after menopause, which can lead to disorders including climacteric syndrome, cardiovascular diseases, and osteoporosis^5,6^. Long-term hormone replacement therapy to prevent these disorders is debated due to adverse effects, such as increased risks of malignancies in reproductive tissues and thromboembolism^7^. To be able to mediate tissue-specific signaling of E2 and avoid adverse effects, we need a comprehensive understanding of how E2 exerts effects in various tissues.

E2 exerts its main effects by binding to estrogen receptors alpha (ERα) or beta (ERβ). It is well-known that in the nucleus, a complex consisting of E2 bound to an ER-dimer can bind to gene promoter regions and regulate transcription. ERα plays a vital role in the nuclear effects, given the fact that ERα-AF2 knockout mice, which lack the activation function 2, are deprived of essentially all the E2-induced transcriptional activities^8^. Beyond effects in the nucleus, studies have shown that rapid extranuclear effects, initiated by ERα located in the membrane (mERα), also play an important role in various cell types^9–11^. In genetically modified mouse models, abrogation of mERα signaling by mutating palmitoylation site C451 in ERα results in phenotypic changes in a tissue-specific way^11,12^. E2 (17β-estradiol) treatment in gonadectomized mice without mERα signaling also reveals a strong tissue-dependent response of E2^13–16^, and this knowledge could be used for searching future therapeutic targets for disorders related to low E2 levels, such as postmenopausal osteoporosis.

It has been shown that mERα signaling can contribute to the regulation of nuclear transcription via a crosstalk between membrane-initiated signaling and nuclear events^17^. Studies have shown that mERα can modulate nuclear effects by affecting downstream signaling of i.e., PI3K/AKT and ERK/MAPK pathways, leading to altered phosphorylation of co-regulatory proteins^18–20^. It is also shown that mERα can interact with other membrane-localized receptors such as epidermal growth factor receptor (EGFR) to influence nuclear effects.

In addition to the tissue-specific E2 treatment effects caused by signaling via mERα, the dose of E2 can also affect the treatment effects in a tissue-specific manner. Studies have revealed that sensitivities to E2 treatment vary between tissues^21,22^. For example, uterus is known to be a highly E2-sensitive organ, while trabecular bone is less sensitive^21^. The E2 treatment studies in mERα-signaling deprived mouse models have so far only investigated one E2 dose each, and the studies vary in administration route, dose, treatment length, gender and/or age^11–16,23^. Some of these studies have shown conflicting results on tissue responses to E2 treatment, however, due to the differences in study designs, it is inappropriate to directly compare E2 sensitivity in different tissues between studies. To the best of our knowledge, no detailed E2 sensitivity studies have been conducted to explore the importance of mERα-initiated signaling in various tissues when treating mice with different doses of E2. The aim of this study is therefore to explore whether mERα-initiated signaling affects the sensitivity to different doses of E2 treatment in various tissues using C451A female mice which lack mERα-initiated signaling.

## Methods

### Animals

The animal experiments were approved by the Ethical Committee for Animal Research in Gothenburg (Göteborgs djurförsöksetiska nämnd; ethic numbers 136–16, 467–19, 4072–22). All experimental procedures and animal handling were performed and reported according to relevant guidelines and regulations, including ARRIVE guidelines. Homozygous C451A mice, with a point mutation at the C451 palmitoylation site in ERα^11,13^, and wildtype (WT) littermates were generated by breeding female ERαC451A^+/-^ mice with male ERαC451A^+/-^ mice. Primers 5ʼ-CTAAACAAGCTTCAGTGGCTCCTAG-3ʼ and 5ʼ-ACCTGCAGGGAGAAGAGTTTGTGGC-3ʼ were applied for genotyping. All animals were housed in a standard animal facility under controlled temperature (22 °C) and photoperiod (12 h light:12 h darkness cycle). Phytoestrogen-free pellet diet (Teklad diet 2016, Envigo) and tap water ad libitum were given to the mice.

### Treatment

Female C451A mice and their WT littermates were ovariectomized (ovx) at the age of three months. Ovx surgery was performed under anesthesia with isoflurane (Baxter Medical AB, Kista, Sweden). Rimadyl (Orion Pharma AB, Animal Health, Sollentuna, Sweden) or Metacam (Boehringer Ingelheim Animal Health, Ingelheim am Rhein, Germany) was given as a post-operative analgesic. Mice were randomly divided into body weight matched treatment groups, vehicle (veh, Miglyol 812, OmyaPeralta GmbH, Hamburg, Germany) or E2 (17β-estradiol-3-benzoate, Sigma-Aldrich, St. Louis, USA).

Three different doses of E2 were applied in this study, including 0.05 μg/mouse/day as low dose, 0.3 μg/mouse/day as medium dose and 6 μg/mouse/day as high dose. Directly after the surgery (for the low dose experiment) or one week after the surgery (for the medium and high dose experiments), the treatment was administrated daily with subcutaneous injections for three consecutive weeks. The different dose experiments were conducted as independent experiments with four treatment groups each (WT-veh, WT-E2, C451A-veh, and C451A-E2). At termination, mice were anesthetized with Ketador/Dexdomitor (Richter Pharma, Wels, Austria/Orion Pharma), bled, and euthanized by cervical dislocation. Thymus and gonadal fat were collected and weighed. Thymus weight data from the medium dose experiment has been previously published^16^. Uterus was collected and divided into halves. One half was dried at 70 °C for 12 h and weighed to retrieve the uterus dry weight, and the other half was used for preparation of RNA (see below). Tibia was dissected, fixated in 4% paraformaldehyde for 2 days and then stored in 70% ethanol for further analysis.

### Steroid concentration measurements

Serum samples were collected after one week and two weeks of treatment in the high dose experiment, and at termination in all three experiments. Serum steroid concentrations were measured by high-sensitivity liquid chromatography-tandem mass spectrometry (LC–MS/MS) as described previously^24^.

### High-resolution microcomputed tomography

High-resolution microcomputed tomography (μCT) analysis was performed on the proximal tibia using the Skyscan 1172 model (medium dose experiment) or the 1275 model (low and high dose experiments) (Bruker MicroCT, Aartselaar, Belgium). The X-ray tube voltage was 50 kV (1172) or 40 kV (1275) and the current was 200 μA. The angular rotation was 180°, the angular increment 0.70° (1172) or 0.40° (1275), and the voxel size was 4.5 μm (1172) or 7 μm (1275) isotropically. The cortical parameters were measured in the diaphyseal region of the tibia starting 5.2 mm (1172) or 5.3 mm (1275) away from the proximal growth plate and extending a further longitudinal distance of 134 µm (1172) or 210 µm (1275) in the distal direction. The trabecular bone distal to the proximal growth plate was selected for analyses within a conforming volume of interest (cortical bone excluded), commencing at 650 μm (1172) or 504 µm (1275) away from the growth plate and extending a further longitudinal distance of 134 μm (1172) or 210 µm (1275) in the distal direction.

### Real-time PCR

mRNA was isolated from uterus using RNeasy Mini Kit (Qiagen, Hilden, Germany). The extracted mRNA was reversely transcribed into cDNA using the High-Capacity cDNA Reverse Transcription kit (Applied Biosystems, Thermo Fisher Scientific, Waltham, MA). Amplifications were performed using the Applied Biosystem StepOnePlus Real-Time PCR System (Thermo Fisher Scientific) and Assay-on-Demand primer and probe sets (Thermo Fisher Scientific) labeled with the reporter fluorescent dye FAM, as well as PowerUp™ SYBR™ Green Master Mix (Applied Biosystems). Multiple genes were chosen as reference genes based on the features of uterus tissue, including *Ppia*, *Gapdh*, *Rpl7*, *Eef2*, *Actb* and 18S ribosomal RNA^25^. In this study, the relative gene expression values were calculated using the ΔΔCt method normalized with a combination of the six reference genes mentioned above, as described before^26^.

The assays used in this study included proliferation-related genes in uterus including lactotransferrin (*Ltf*: Mm00434787_m1) and cytokeratin 8 (*Krt8*: Mm04209403_g1), apoptosis-related genes including B cell leukemia/lymphoma 2 (*Bcl2*: Mm00477631_m1), TNF receptor superfamily member 6 (*Fas*: Mm01204974_m1), and Fas ligand (*Fasl*: Mm00438864_m1), and reference genes glyceraldehyde-3-phosphate dehydrogenase (*Gapdh*: 4352339E), actin beta (*Actb*: 4352341E), eukaryotic translation elongation factor 2 (*Eef2*: Mm01171435_gH), and 18S (4310893E). Primers used in this study included *Ppia* (PrimerBank-MGH-PGA ID: 6679439a1) and *Rpl7* (PrimerBank-MGH-PGA ID: 31981515a1)^27^. RNA expression data of *Ltf* and *Krt8* in the medium dose experiment was published before^16^, however in this study, new cDNA was synthesized together with the low and high dose experiments.

### Statistical analysis

All values are presented as mean ± SEM. To evaluate the differences in % E2 responses between WT and C451A mice in each individual dose experiment, we applied the interaction factor from two-way ANOVA analysis. Šidák´s multiple comparisons test was used to compare the difference between veh and E2 treatment in WT and C451A mice within each dose experiment (GraphPad Prism version 9.4.1). To compare the E2 responses in various tissues between the different dose experiments (Fig. 3), the calculated % E2 response in WT mice in the high dose experiment was set to 100%. All other % E2 responses were then related to the % E2 response in WT mice from the high dose experiment to visualize the changes in % E2 response between different doses in each tissue (Microsoft Excel). Logarithmic transformations were used when appropriate to ensure normal distribution of data, checked by the Shapiro–Wilk test. A difference was considered significant when *p* < 0.05.

## Results

### E2 treatment by subcutaneous injection results in stable serum E2 concentrations throughout the experiment

**Figure 1:**
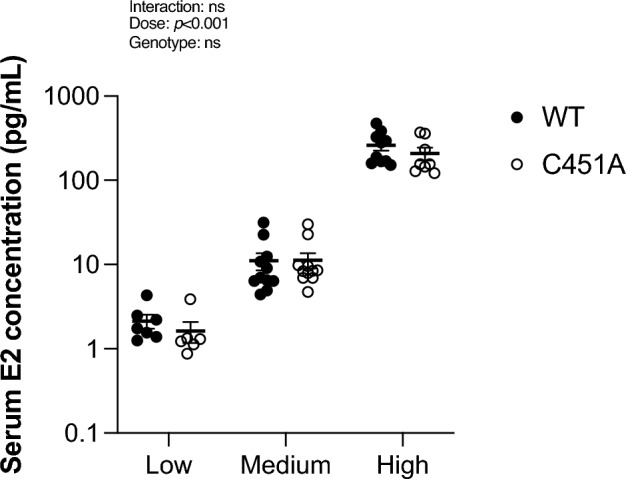
WT and C451A mice have similar serum E2 concentrations in each dose experiment. Ovariectomized WT and C451A females were treated with estradiol (E2) or vehicle (veh) for three weeks. Serum E2 concentration at termination was measured by LC–MS/MS in WT and C451A mice treated with a low dose (0.05 µg/mouse/day; WT, n = 7; C451A, n = 6), medium dose (0.3 µg/mouse/day; WT, n = 11; C451A, n = 11) or high dose (6 µg/mouse/day; WT, n = 10; C451A, n = 8) of E2. Two-way ANOVA followed by Šidák´s multiple comparisons test was applied for analysis. All individual values are presented with mean (horizontal line) and SEM (vertical lines). ns; not significant.

By daily subcutaneous injections of E2, we managed to achieve stable serum concentrations of E2 over time (Suppl. Table 1), and the concentrations did not differ between WT and C451A females (Fig. 1). Based on the serum E2 concentrations in E2-treated mice at termination, we named the three experiments as low dose (≈ 2 pg/ml in serum, corresponding to a low physiological concentration), medium dose (≈ 11 pg/ml in serum, corresponding to a high physiological concentration), and high dose (≈ 237 pg/ml in serum, corresponding to a clearly supraphysiological concentration)^28^.

### Loss of mERα signaling results in decreased sensitivity to E2 treatment

**Figure 2:**
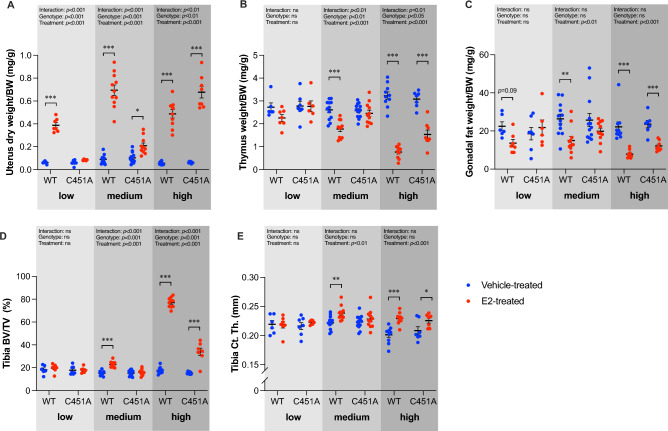
Loss of mERα signaling reduces the sensitivity to E2 treatment in a dose- and tissue- dependent manner. Ovariectomized WT and C451A females were treated with vehicle (veh) or estradiol (E2) for three weeks. Low dose (0.05 µg/mouse/day; WT, n = 7; C451A, n = 6), medium dose (0.3 µg/mouse/day; WT, n = 11; C451A, n = 11) or high dose (6 µg/mouse/day; WT, n = 10; C451A, n = 8). Relative tissue weights, normalized against body weight (BW), of dried uterus (**A**), thymus (**B**), and gonadal fat (**C**). Trabecular bone volume per tissue volume (BV/TV) (**D**) and cortical thickness (Ct. Th.) (**E**) of tibia were analyzed by high-resolution microCT. Two-way ANOVA followed by Šidák´s multiple comparisons test was applied for analysis. All individual values are presented with mean (horizontal line) and SEM (vertical lines). *p < 0.05, **p < 0.01, ***p < 0.001, ns; not significant.

In WT mice, uterus was the most sensitive target of E2 given the fact that the low dose of E2 was adequate to increase uterus weight, while none of the other examined tissues were significantly affected (Fig. 2). However, in C451A mice, which lack mERα signaling, a low dose of E2 failed to trigger any effect in uterus as well as in all the other tissues (Fig. 2).

With the medium dose of E2, all tissues manifested textbook-like effects of E2 in WT mice, including increased weight of uterus, decreased weights of thymus and gonadal fat, and increased trabecular bone mass and cortical thickness in tibia (Fig. 2). In the absence of mERα signaling, these effects were gone, except for an increased uterus weight (Fig. 2A). However, the increase in uterus weight was significantly reduced in C451A mice compared to the increase in WT mice with this medium stimulation of E2 (− 85%,* p* < 0.001).

With a supraphysiological E2 treatment, all the examined tissues in both WT and C451A mice showed explicit E2 effects (Fig. 2). For gonadal fat weight and cortical thickness, the high concentration of E2 was able to largely compensate for the loss of mERα signaling (Fig. 2C,E), since there were no significant differences in the E2 responses between WT and C451A mice. In contrast, the high concentration of E2 failed to fully compensate for the loss of mERα signaling in thymus and trabecular bone mass (Fig. 2B,D), implicating a more prominent importance of mERα-initiated signaling in regulating E2 effects in these tissues. Unexpectedly, the estrogenic response to high-dose E2 treatment on uterus weight was increased in C451A mice compared to WT mice (Fig. 2A).

### Loss of mERα signaling causes a right shift of E2 responses

**Figure 3:**
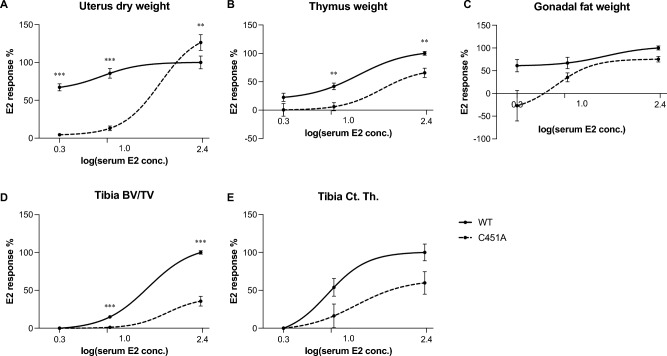
Loss of mERα signaling causes a right shift of E2 treatment responses. Tissue responses to different doses of E2 treatment in uterus (**A**), thymus (**B**), gonadal fat (**C**), tibia trabecular bone (**D**), and tibia cortical bone (**E**). X-axis represents the logarithmic mean value of serum E2 concentrations at termination (pg/mL). Y-axis represents the % response to E2 treatment calculated from % E2 response in WT mice in the high-dose experiment, which was set to 100%. All other % E2 responses were related to the % E2 response in WT mice from the high-dose experiment. The E2 response curve is visualized by using a sigmoidal, four parameter logistic (4PL) curve fitting model (GraphPad). Analyses of differences in E2 responses between WT and C451A were done by two-way ANOVA. All individual values are presented with mean (horizontal line) and SEM (vertical lines). **p < 0.01, ***p < 0.001, conc.; concentration, BV/TV; bone volume per tissue volume, Ct. Th.; cortical thickness.

As shown in the E2 dose response curves in Fig. 3, there are in general obvious right-shifts of E2 responses in C451A mice compared to WT mice in all investigated tissues. In the absence of mERα signaling, treatment with a high dose of E2 managed to rescue the E2 effects on uterus weight (Fig. 3A), and even result in higher E2 response in C451A mice compared to WT mice (126% of the E2 response in WT mice). However, in thymus and trabecular bone, notwithstanding the high dose, E2 was not able to rescue the blunted responses caused by the loss of mERα signaling (Fig. 3B,D). In contrast, E2 responses in gonadal fat and cortical bone were not significantly changed between WT and C451A mice (Fig. 3C,E).

### Loss of mERα signaling affects proliferation-related and apoptosis-related genes in uterus

In an attempt to determine the underlying mechanism of how different doses of E2 and the loss of mERα signaling can affect the uterus weight, we measured mRNA expression of proliferation (*Ltf* and *Krt8*) and apoptosis (*Bcl2*, *Fas*, and *FasL*) related genes in uterus.

**Figure 4:**
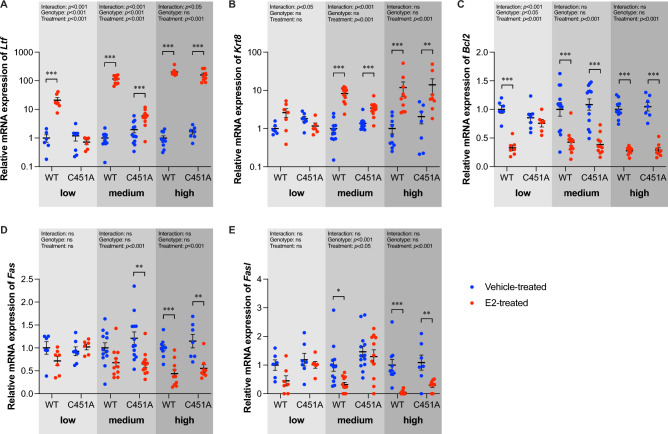
Loss of mERα signaling affects proliferation-related and apoptosis-related genes in uterus. Relative mRNA expression of proliferation-related genes in uterus, including *Ltf* (**A**) and *Krt8* (**B**), and apoptosis-related genes, including *Bcl2* (**C**), *Fas* (**D**) and *Fasl* (**E**) in ovx WT and C451A females treated with estradiol (E2) or vehicle (veh) for three weeks. Low dose (0.05 µg/mouse/day; WT, n = 7; C451A, n = 6), medium dose (0.3 µg/mouse/day; WT, n = 11; C451A, n = 11) or high dose (6 µg/mouse/day; WT, n = 10; C451A, n = 8). Two-way ANOVA followed by Šidák´s multiple comparisons test was applied for analysis. All individual values are presented with mean (horizontal line) and SEM (vertical lines). *p < 0.05, **p < 0.01, ***p < 0.001, ns; not significant.

As shown in Fig. 4A,B, the expression of *Ltf* was significantly increased, while *Krt8* was unaffected, in WT mice treated a with a low dose of E2. By increasing the dose of E2 to medium and high doses, the mRNA expressions of both *Ltf* and *Krt8* were upregulated in WT mice (Fig. 4A,B). In C451A mice, neither of the proliferation-related genes was significantly affected by a low stimulation of E2 (Fig. 4A,B). When given a medium dose of E2, both genes were upregulated in C451A mice, but the effects remained significantly reduced compared to the effects in WT mice (Fig. 4A,B). When given a supraphysiological stimulation of E2, the expression of *Krt8* was similarly increased in C451A and WT mice (Fig. 4B), but the E2 effect on *Ltf* expression was still slightly, but significantly, blunted in C451A mice compared to WT mice (Fig. 4A).

The anti-apoptosis gene *Bcl2* was not affected by treatment with a low dose of E2 in C451A mice, whereas it was downregulated in WT mice (Fig. 4C). However, when treated with medium and high doses of E2, the expression of *Bcl2* was similarly decreased in WT and C451A mice, suggesting that the regulation of *Bcl2* is independent of mERα signaling after medium and high dose E2 treatment, while mERα-dependent when the E2 dose is low (Fig. 4C). The classic pro-apoptosis pathway genes *Fas* and *Fasl* were not affected in either WT or C451A mice after treatment with low dose E2 (Fig. 4D,E). With a medium dose of E2, *Fas* was downregulated in C451A mice and *Fasl* was downregulated in WT mice (Fig. 4D,E). With a high dose of E2, WT and C451A mice showed same levels of downregulation of *Fas* and *Fasl* gene expressions (Fig. 4D,E).

## Discussion

Estrogen signaling leads to several beneficial effects throughout the body, including increased bone mass and relief of menopausal symptoms^29^. However, since estrogen signaling is also associated with unwanted adverse effects, such as an increased risk of malignancy in reproductive tissues^30^, more knowledge regarding the mechanism underlying tissue-selective effects of E2 is required to develop treatments with fewer adverse effects. It is well-proven that the sensitivity to E2 treatment in mice varies between tissues^21,22^. Furthermore, we and others have previously demonstrated that the dependence of mERα signaling for the responses to E2 treatment varies in a tissue-specific manner^12–14,16^. To determine whether mERα signaling affects the sensitivity to E2 treatment differently in different E2-sensitive tissues, we treated mice lacking mERα signaling with different doses of E2 and showed that loss of mERα signaling in general decreases the sensitivity to E2 treatment in a dose- and tissue- dependent manner.

Several studies in which C451A mice are treated with E2 have been published^11–16,23^. However, the results have been somewhat inconsequent and shown differences regarding the dependency of mER signaling in various non-reproductive E2 responsive tissues and uterus. For example, in Gustafsson et al.^13^, E2 treatment of female ovx C451A mice resulted in significant effects in thymus and bone, while no E2 treatment effects in these tissues were detected in ovx C451A mice in Gustafsson et al.^16^. Interestingly, the E2 serum concentration after treatment with slow-release pellet (0.001 mg for a 60-day release, which should provide 0.02 μg/mouse/day), used in Gustafsson^13^, was 36.7 ± 2.1 pg/mL (unpublished data), while treatment with subcutaneous injections (0.3 μg/mouse/day), used in Gustafsson et al.^16^, resulted in a lower serum concentration at termination, 11.3 ± 2.3 pg/mL (unpublished data). These results regarding E2 concentrations are not that surprising since subcutaneous slow-release pellets are reported to produce an early and extremely supraphysiological E2 peak, which subsequently declines^31,32^ (same releasing peak was detected in our laboratory, unpublished data). Thus, it was expected from previous work that the dependence of mERα signaling might be dose-dependent, but the results are difficult to compare due to differences in administration routes, doses, treatment length, gender, and/or age. Therefore, in this study we applied daily subcutaneous injections of different E2 doses, which resulted in stable serum concentration of E2 throughout the experiments.

The sensitivity to E2 treatment is clearly dependent on mERα-initiated signaling, given the fact that C451A mice responded poorly to both low and medium doses of E2 treatment. Thus, when serum E2 concentrations are within the physiological range^28^, mERα signaling is indispensable for the effects in bone, thymus, and gonadal fat, while the uterus can still respond even when mERα is absent. In contrast, treatment with a supraphysiological dose of E2 resulted in significant effects in all tissues examined, clearly demonstrating that increasing the dose of E2 can circumvent the dependence of mERα signaling. Interestingly, when compared to WT littermates, C451A mice showed significantly reduced responses to high dose E2 treatment in thymus and trabecular bone, while the E2 treatment effects in adipose tissue, cortical bone and uterus were not blunted. These results show that the desensitization to E2 treatment at a supraphysiological dose, caused by the loss of mERα signaling, has an obvious tissue-specific pattern. It is difficult to know if the E2 response would be fully rescued in thymus and trabecular bone by an even higher dose of E2, since higher doses can be harmful to the mice and therefore it is not ethical to conduct such experiment. Interestingly, the dependence of mERα signaling in the high-dose experiment differed between the trabecular and cortical bone compartments. Previous animal studies have shown that cortical and trabecular bone are regulated differently in many settings^33–35^. This study further demonstrates an important role for mERα-initiated signaling in mediating the diversity between the two bone compartments.

It has previously been shown that uterus is a highly E2-sensitive tissue^16,21^, and in this study, the uterus was also by far the most E2-sensitive of the tissues examined. It is very interesting to find out that the mERα-dependency for the E2 treatment effects in uterus varies when treating with different doses of E2. A low dose of E2 failed to trigger any effect on uterus weight in C451A mice, while the medium dose induced a significant, but still blunted, effect in C451A mice compared to WT mice. Interestingly, supraphysiological stimulation of E2 resulted in a higher effect in C451A mice compared to the effect in WT mice. It has previously been shown in WT mice that the E2 sensitivity in uterus is reduced after high E2 treatment compared to treatment with a lower E2 dose^22^, and one possibility is that the loss of mERα signaling somehow blunts this reduction in E2 sensitivity. In an attempt to understand the mechanism behind the dose-dependent effects caused by the loss of mERα signaling, we measured RNA expression of several proliferation and apoptosis related genes in the uterus. Under low and medium doses of E2, the expression of the proliferative genes *Krt8* and *Ltf* were quite coherent with the uterus phenotypes, showing a significant upregulation in WT mice compared to C451A mice. Treatment with a supraphysiological E2 dose resulted in a similar upregulation of the expression levels of *Krt8* and *Ltf* between WT and C451A, indicating that the increased response in uterus in C451A mice compared to WT mice might not be caused by an increase in proliferation. Apoptosis-related genes were also investigated, and high dose E2 treatment decreased the expression of *Bcl2*, as well as the expressions of *Fas* and *Fasl* to a similar extent in WT and C451A mice. Thus, the increased uterine weight in response to a high dose of E2 in C451A mice compared to WT mice cannot be explained by regulation of these specific apoptosis-related genes. Further studies are needed to fully understand the mechanisms of how mERα enhances the E2 effect on uterus weight when using physiological E2 treatment while inhibiting the response to supraphysiological E2 treatment. There are also several in vitro studies indicating that nongenomic ERβ signaling can modulate E2 effects in different cells^36–38^. Further in vivo studies regarding the role of nongenomic ERβ signaling for uterine effects is thus warranted in the future.

Previous studies have reported conflicting results regarding the mERα dependency for the E2 treatment effect on uterus weight^12,16^. In Gustafsson et al.^16^, when giving 3-month-old ovariectomized mice a physiological dose of E2, the uterus response was clearly mERα dependent^16^. In contrast, treatment of younger mice, ovariectomized at 1 month of age, with slow-releasing E2 pellets, resulted in a uterus response that was clearly mERα independent^12^. The two studies differ regarding the age of the mice, however, based on the results presented in the current study, we propose that the main cause of the differences in the uterine response between these two studies is the E2 concentration. Adlanmerini et al. used a pellet treatment that has been reported to result in higher E2 concentrations than expected^32^, and this might have resulted in an E2 concentration that is able to circumvent the mERα dependency.

**Figure 5:**
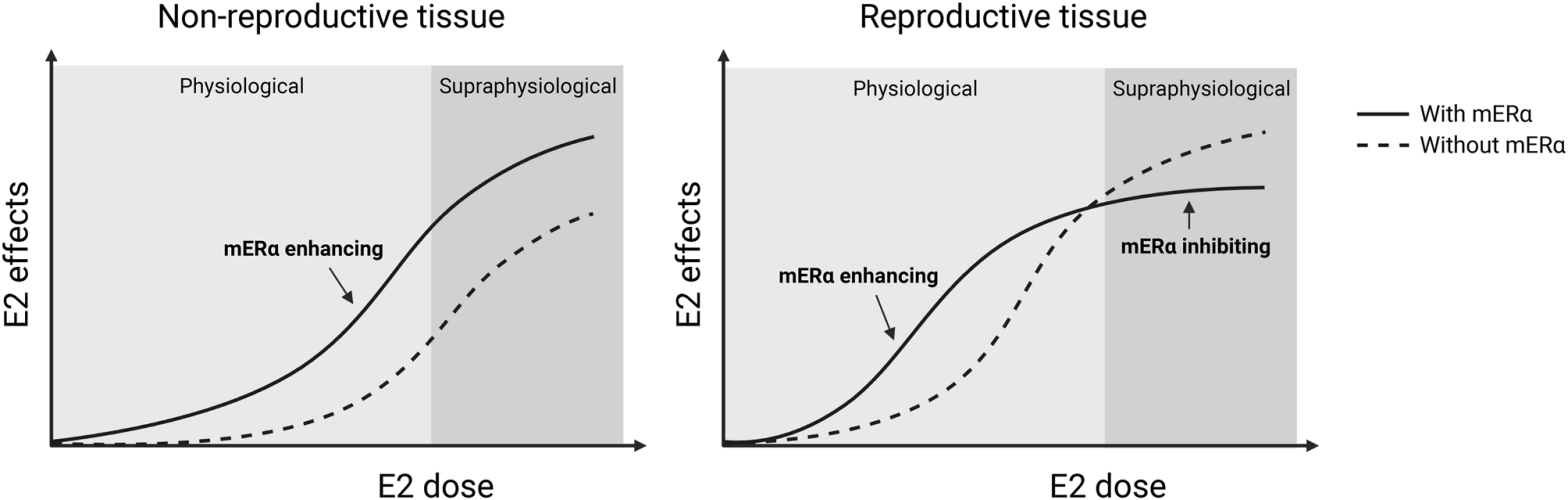
Proposed role of mERα-initiated signaling after treatment with physiological and supraphysiological doses of E2. In this study, it has been shown that the sensitivity to physiological E2 treatment is reduced in both non-reproductive tissues and uterus in the absence of mERα signaling. However, with a supraphysiological E2 dose, the effect in uterus is enhanced when mERα signaling is lacking. Therefore, we suggest that mERα signaling in general enhances E2 effects within the physiological range, and that it may also partially protect the uterus from potential harm of supraphysiological levels of E2. Created with BioRender.com.

Based on the findings in the present study, we propose that mERα signaling in general enhances the estrogenic response to physiological E2 treatment in both non-reproductive tissues and uterus (Fig. 5). However, mERα signaling also has the capacity to inhibit the estrogenic response to supraphysiological E2 treatment on uterus weight (Fig. 5). One may speculate that this mechanism may partly protect the uterus from harmful supraphysiological concentrations of E2.

We have previously shown that the dependence of mERα signaling differs between tissues after treatment with selective estrogen receptor modulators (SERMs)^16^. Previous studies have also shown that different doses of SERMs can have various effects in different tissues^39–41^. An appropriate dose of a SERM that can manipulate mERα signaling might result in less adverse effects. Therefore, investigating the importance of mERα signaling for the sensitivity of SERMs under different doses will be an important task that might aid the development of better SERM treatments in the future.

To conclude, this study shows that loss of mERα-initiated signaling reduces sensitivity to physiological E2 treatment in both non-reproductive tissues and uterus. However, the effect of supraphysiological E2 treatment in uterus is enhanced in the absence of mERα. We therefore propose that mERα signaling in general enhances the effects of E2 in the physiological range but that mERα signaling may also have the capacity to partly protect the uterus from harmful supraphysiological levels of E2.

## Competing interests

C.O. has two patents/patent applications in the field of probiotics and bone health. All other authors declare that they do not have any competing interest.

## Supplementary Information

Supplementary Table 1.

## Data Availability

The data that support the findings of this study are available in the methods, results, and supplementary material of this article, and in figshare repository, https://figshare.com/s/317b9aa288956fec79fc.
