# Supplementary material for: Membrane estrogen receptor α signaling modulates the sensitivity to estradiol treatment in a dose- and tissue- dependent manner
Source: Sci Rep. 2023 Jun 3;13:9046. doi: 10.1038/s41598-023-36146-9 (PMC10239518; doi:10.1038/s41598-023-36146-9)
Supplement: Supplementary file 1 — Supplementary Table 1. [file 41598_2023_36146_MOESM1_ESM.pdf]

Membrane estrogen receptor α signaling modulates the sensitivity to estradiol treatment in a dose- and tissue- dependent manner

Yiwen Jiang[1#], Karin Horkeby[1#*], Petra Henning[1], Jianyao Wu[1], Lina Lawenius[1], Cecilia Engdahl[1,3], Priti Gupta[1,3], Sofia Movérare-Skrtic[1], Karin H Nilsson[1], Ellis Levin[4], Claes Ohlsson[1,2], Marie K Lagerquist[1]

Supplementary Table 1. Stable serum E2 levels in high-dose E2 treated groups throughout the experiment.

Timepoint	WT-E2	C451A-E2	Šídák's multiple comparisons test
**1-week**	184±13 (n=6)	193±29 (n=5)	ns
**2-week**	226±20 (n=5)	330±32 (n=3)	ns
**3-week**	260±33 (n=10)	209±34 (n=8)	ns

(Two-way ANOVA analysis: interaction: ns; timepoint: ns; genotype: ns)

Ovariectomized WT and C451A females were treated with 17beta-estradiol (E2) of 6 µg/mouse/day for three weeks. Serum samples were taken after 1-week, 2-week and 3-week of treatment, and serum E2 levels were measured by LC-MS. Two-way ANOVA followed by Šidák´s multiple comparisons test was applied for analysis. All values are presented as mean ± SEM. ns; not significant.
